# Targeting the Insulin-Like Growth Factor 1 Receptor in Ewing's Sarcoma: Reality and Expectations

**DOI:** 10.1155/2011/402508

**Published:** 2011-05-03

**Authors:** David Olmos, Ana Sofia Martins, Robin L. Jones, Salma Alam, Michelle Scurr, Ian R. Judson

**Affiliations:** ^1^Sarcoma Unit, The Royal Marsden NHS Foundation Trust, London SW3 6JJ, UK; ^2^Sarcoma Molecular Pathology Team, The Institute of Cancer Research, Sutton SM2 5N6, UK; ^3^Drug Development Unit, The Royal Marsden NHS Foundation Trust, Downs Road, Sutton SM2 5PT, UK; ^4^Fred Hutchinson Cancer Research Center, Seattle, WA 95109-4433, USA

## Abstract

Ewing's sarcoma family of tumours comprises a group of very aggressive diseases that are potentially curable with multimodality treatment. Despite the undoubted success of current treatment, approximately 30% of patients will relapse and ultimately die of disease. The insulin-like growth factor 1 receptor (IGF-1R) has been implicated in the genesis, growth, proliferation, and the development of metastatic disease in Ewing's sarcoma. In addition, IGF1-R has been validated, both *in vitro* and *in vivo*, as a potential therapeutic target in Ewing's sarcoma. Phase I studies of IGF-1R monoclonal antibodies reported several radiological and clinical responses in Ewing's sarcoma patients, and initial reports of several Phase II studies suggest that about a fourth of the patients would benefit from IGF-1R monoclonal antibodies as single therapy, with approximately 10% of patients achieving objective responses. Furthermore, these therapies are well tolerated, and thus far severe toxicity has been rare. Other studies assessing IGF-1R monoclonal antibodies in combination with traditional cytotoxics or other targeted therapies are expected. Despite, the initial promising results, not all patients benefit from IGF-1R inhibition, and consequently, there is an urgent need for the identification of predictive markers of response.

## 1. Introduction

Sarcomas represent a diverse group of tumours that arise from connective tissue, and account for 12% of paediatric malignancies and approximately 1% of all adult tumors [1–3]. Significant progress has been made in the classification, staging, and multimodal treatment of these heterogeneous conditions including: surgical advances in functional preservation, the use of radiotherapy as adjunct to other modalities, and the identification of active systemic therapies for certain sarcoma subtypes [4–6]. 

Ewing's sarcoma family of tumours (ESFTs) comprise an aggressive group of sarcomas which can arise in soft tissue or bone and include classical Ewing's sarcoma, Askin tumour, and primitive neuroectodermal tumour (PNET) [7]. These tumours are most commonly diagnosed in adolescence [8], although increasing numbers are being identified in adults [9], have a slight male predominance, and are more common in Caucasian populations [8, 9]. Approximately a quarter of patients present with metastatic disease at the time of diagnosis [10], and the most common metastatic sites are lung (50%), bone (25%), and bone marrow (20%). Over the last 30 years, the prognosis for patients with localised disease has improved dramatically. The introduction of combination chemotherapy has improved survival from 20%–30% (with surgery alone) to 60%–70% with multi modality management [11]. Yet, the prognosis for patients with metastatic or recurrent disease is very poor with only 30%–40% achieving a cure. Furthermore, the prognosis for relapsing patients is very poor, with a 5-years survival rate between 20% to 25% [12], and it is even worse in those who relapse during the first 24 months [13, 14]. Therefore, there is an urgent need for developing better therapies to treat these patients with very poor prognosis.

The ESFTs family is characterised by specific chromosomal translocations involving the fusion of the *EWS* gene and *ETS* family of transcription factors. The translocation *t*(11;22) which results in the *EWS-FLI1* fusion gene is the commonest [15]. Work by Scotlandi and colleagues revealed that IGF-1R system was activated in Ewing's sarcoma cell lines and tumours by an autocrine loop [16]. Subsequently, Prieur and colleagues demonstrated the potential role of the* EWS-FLI1 *fusion protein in Ewing's sarcoma in the IGF-1R pathway activation by repressing IGF-binding proteins [17]. The aim of this manuscript is to review the preclinical and clinical data supporting the use of IGF-1R inhibitors in ESFTs.

## 2. The IGF-1R Pathway

**Figure 1:**
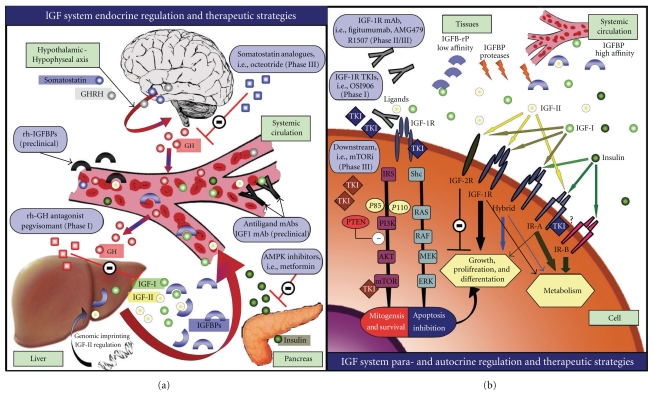
The endocrine, paracrine and autocrine regulation of the IGF-1R pathway and therapeutic strategies for its disruption. (a), Systemic regulation at the endocrine level. The GH-IGF-IGFBP is directed by the hypothalamus-hypophysis axis, where GH is produced, and mediated by the hypothalamus GH releasing factors (which include GHRH and somatostatins). Disruption of the hypothalamus and hypophysis axis, and thus GH release inhibition, has been attempted with somatostatin analogues (octeotride) in a Phase III trial [18]. However, this trial failed to meet the endocrinological and clinical endpoints. Pegvisomant (Pfizer) a human recombinant GH receptor antagonist, has been tested successfully for the treatment of acromegaly [19]. This pegylated recombinant human analogue of GH can decrease the production and release of IGF-I. Other strategies in preclinical development resulting in the reduction of the proportion of free ligand include antiligand mAbs [20] or recombinant IGFBPs. (b) Free-ligand levels at tissue level are also regulated by the presence of the different IGFBPs. This figure illustrates the downstream signalling cascades that result in stimulation of the cell cycle and translation, leading to increased proliferation and growth and inhibition of apoptosis. The IGF-1R pathway can be disrupted by using anti-IGF-1R mAbs and tyrosine kinase inhibitors (TKIs). Another potential strategy is represented by the inhibition of downstream intracellular tyrosine kinase proteins, that is, multiple small molecule inhibitors against PI3K, AKT, RAF, MEK, and mTOR inhibitors [21]. (Adapted from [22]).

The IGF-1R pathway is more than a simple growth factor receptor, its ligands and a downstream signalling cascade. In vertebrates, it plays a key role in the growth and development of normal tissues and regulates the overall growth of organisms [23–25]. This pathway is also part of a more complex insulin-related signalling network. In the evolutionary process, the insulin-like growth factor receptors and IGF system have developed from a single, common ancestral receptor [26, 27] to a more complex system which involves three ligands (IGF-I, IGF-II, and insulin) and at least four receptors (IGF-1R, IGF-IIR, the insulin receptor (IR), and hybrid receptors) [28]. A diagram of the endocrine, paracrine, and autocrine regulation of this pathway is represented in Figure 1.

The IGF-1R is a transmembrane receptor that is activated by IGF-1 and by the related growth factor IGF-2. It is a tetrameric transmembrane receptor tyrosine kinase composed of two *α* and two *β* subunits linked by disulfide bonds. The extracellular *α* subunit is responsible for ligand binding, whereas the *β* subunit consists of a transmembrane domain and a cytoplasmic tyrosine kinase domain [29, 30]. The receptor is primarily activated by its cognate ligands, insulin-like growth factor I (IGF-I) and II (IGF-II; 2- to 15-fold lower affinity), and to a much lower affinity by insulin. The ligands bind to the cysteine-rich domain of the *α*-subunits, leading to the transmission of a signal through the transmembrane domain to the *β*-subunit. The *β*-subunit responds by undergoing a conformational change that causes stimulation of tyrosine kinase activity, followed by autophosphorylation of a cluster of tyrosine residues of the IGF-1R [31]. Then, IGF-1R activates alternative pathways for protection from apoptosis, cell proliferation, and differentiation. One of these pathways leads to the activation of PI3K-AKT-mTOR, while another pathway results in MAPKs (mitogen-activated protein kinases) activation. All these pathways, however, result in maintenance of cell survival by antagonizing the processes and proteins involved in apoptosis. This multiplicity of signalling pathways used by the IGF-1R may explain why this receptor has such powerful and widespread antiapoptotic activity [32–34].

## 3. Biological Implication of the IGF-1R Pathway in Ewing's Sarcoma

The involvement of the IGF system in sarcoma initiation and progression has been associated with postnatal development [35, 36], primarily in those tumours that occur in younger patients. During this growing period, the function of growth hormone (GH) is mediated by IGF1. This is important, since IGFs are important regulators of growth and development in normal bone, contributing to about 50% of basal bone-cell proliferation [37]. Therefore, overexpression of genes involved in GH or IGF signalling may favour cell growth, thus increasing the risk of tumorigenesis.

In the case of Ewing's sarcoma, IGF-1R is ubiquitously expressed and its activation is sustained by the autocrine production of IGF1 by tumour cells [38, 39]. *In vitro* studies have shown that IGF-1R is directly involved in Ewing's sarcoma cell proliferation and survival [16, 40–42]. It has also been shown that *EWS-FLI1*, the genetic hallmark of Ewing sarcoma, is only capable of transformation in the presence of IGF-1R [43] and, more recently, that this fusion product directly affects IGF-1R signalling either by downregulating IGFBP3 [17], increasing IGF1 promoter, or both [44]. Additionally, it has been shown that forced expression of EWS-FLI in mesenchymal stem cells resulted in transformation into a phenotype similar to Ewing's sarcoma. The cells transformed by the fusion gene expressed high levels of IGF1 and were dependent on IGF-1R signalling for growth and survival [45]. Similarly, in mouse fibroblasts, IGF-1R expression was necessary for *EWS-FLI*-mediated transformation [43]. These *in vitro* results have been confirmed with the finding of IGF-1R expression in clinical samples of Ewing's sarcoma and the demonstration that lower levels of IGF-1R expression correlate with a lower tumor proliferative rate and a better prognosis [46]. However, the limitations of this study in terms of its retrospective nature and the antibodies used should be noted. Despite such limitations, this observation is important when planning clinical trials, where stratification of patients for biological variables may be important. 

The evidence described above supports a role for drugs targeting IGF-1R signalling in Ewing's sarcoma. Blockade of IGF-1R has been shown to cause inhibition of cancer cell proliferation, survival, and anchorage-independent growth *in vitro*, to inhibit tumourigenesis, and block tumour invasion and metastasis, and to sensitize cancer cells to chemotherapy and radiotherapy [47].

## 4. Preclinical Experience Targeting IGF-1R in Ewing's Sarcomas

Despite the advances in the treatment of Ewing's sarcoma, many patients still succumb due to the development of metastatic or recurrent disease, and there is recognition that the benefit achieved with conventional cytotoxic therapy has reached a plateau. The need to identify and validate biologically critical targets is, therefore, extremely urgent. To achieve this aim, a large number of targeted therapeutic approaches have been evaluated in Ewing's sarcoma models, both *in vitro* and *in vivo*. Some of these targets, including IGF-1R, have been validated in preclinical studies and IGF-1R inhibitors are currently undergoing evaluation in clinical trials. Among the various strategies used to interfere with IGF-1R function in preclinical studies, monoclonal antibodies (mAbs) and small molecule tyrosine kinase inhibitors represent the best candidates for clinical development. 

Monoclonal antibodies need the following properties to be effective: they must inhibit binding of IGF1 and IGF2, induce receptor downregulation, and have little or no effect on insulin receptor signalling. Promising *in vitro* and *in vivo* studies have shown antitumor activity of several mAbs, resulting in inhibition of proliferation, apoptosis induction, and tumour growth inhibition [16, 48, 49]. 

There are a number of oral small molecule tyrosine kinase inhibitors in development. *In vitro* studies with a number of these agents have demonstrated inhibition of IGF-1R, high level of growth inhibition, survival reduction, complete pathway blockade, and xenograft tumor growth reduction [41, 50–52]. However, receptor downregulation was not observed with tyrosine kinase inhibitors, and this may partly account for their cytostatic, rather than cytotoxic effects against Ewing's sarcoma xenografts [53]. 

Whether or not complete IGF-1R selectivity should be achieved is still under debate. Depending on the mechanism, inhibition of IGF-1R may target not only IGF-1R itself but also the hybrid receptors (especially those containing the fetal isoform insulin receptor-A) which favour cancer cell proliferation and are activated by both IGFs. It has been shown that targeting IGF-1R increases the efficacy of other anticancer therapies. This is based on evidence that IGF-1R signalling protects tumour cells from many insults, including chemotherapeutic agents and ionizing radiation [54–56], thus limiting the efficacy of such therapy. Inhibition of IGF-1R signalling has been shown to increase the sensitivity of Ewing's sarcoma cells to chemotherapy [51, 57, 58]. Combining IGF1-R with conventional therapy may have the advantage of lowering the effective dosage of radiotherapy and chemotherapy, minimizing side effects while maintaining efficacy. This is particularly important for paediatric patients. In addition to a potential role in combination with traditional cytotoxic regimens and with radiotherapy, there are data demonstrating involvement of IGF-1R in trastuzumab resistance [59, 60] and resistance to AKT/mTOR inhibitors [61]. It has been shown that IGF-1R blockade can restore sensitivity to these agents.

An important issue in developing agents that specifically target IGF-1R is its high level of homology with the insulin receptor. There is a complete homology at the ATP-binding pocket and 84% homology within the intracellular kinase domain [62]. It is important to determine not only overlapping but also different biological effects of both receptors. Although both similarly activate PI3K and MAPK pathways [63, 64], subtle differences exist in the recruitment of certain docking proteins and intracellular mediators. These differences may be exploitable in terms of developing specific IGF-1R inhibitors. However, currently, there are no published data specifically addressing the role of the insulin receptor in Ewing's sarcoma.

## 5. Clinical Experience with IGF-1R Targeted Treatments in Ewing's Sarcoma

At the time of this review, mAbs against IGF-1R represent the most clinically advanced means of inhibiting this pathway in the treatment of Ewing's sarcoma patients. Several antibodies have been tested in Phase II studies. Other approaches for blocking or disrupting IGF-1R activity in Ewing's sarcoma patients include (a) the reduction of ligand levels or bioactivity or (b) the inhibition of receptor function using small-molecule tyrosine-kinase inhibitors [82]. Examples of different strategies for targeting the IGF-1R pathway are represented in Figure 1.

Several anti-IGF-1R mAbs have been developed for clinical use through the humanization of mouse mAbs, immunization of genetically engineered mice that produce fully human antibodies, or the selection of specific antibodies from phage display libraries. These antagonistic IGF-1R mAbs work through two major mechanisms: first by immediate inhibition of ligand binding, and secondly by a delayed effect on the downregulation of IGF-1R. At present, eight different mAbs have been evaluated in clinical trials: figitumumab (CP-751,871), ganitumab (AMG479), robatumumab (R1507), cixutumumab (IMC-A12), dalotozumab (MK0646), SCH-717454, AVE-1642, and BIIB-022. Other reviews have extensively discussed the differences and similarities of these antibodies [22, 83]. In general, these mAbs are IgG1 isotype [65, 73, 84–87] with the exception of figitumumab and BIIB022 which are IgG2 [88] and IgG4 [71] isotype, respectively. There are significant pharmacokinetic and immunologic differences between IgG1, IgG2 and IgG4 isotypes. IgG2 mAbs appear to have longer half-lives than IgG1 and IgG4 mAbs, while IgG1 mAbs are usually potent activators of the classical complement pathway, complement-dependent cell-mediated cytotoxicity and antibody-dependent cellular cytotoxicity [89].  Table 1 reviews all the IGF-1R antibodies in clinical development.

### 5.1. Early Clinical Studies with Anti-IGF-1R Mabs Involving Ewing Sarcoma Patients

**Figure 2:**
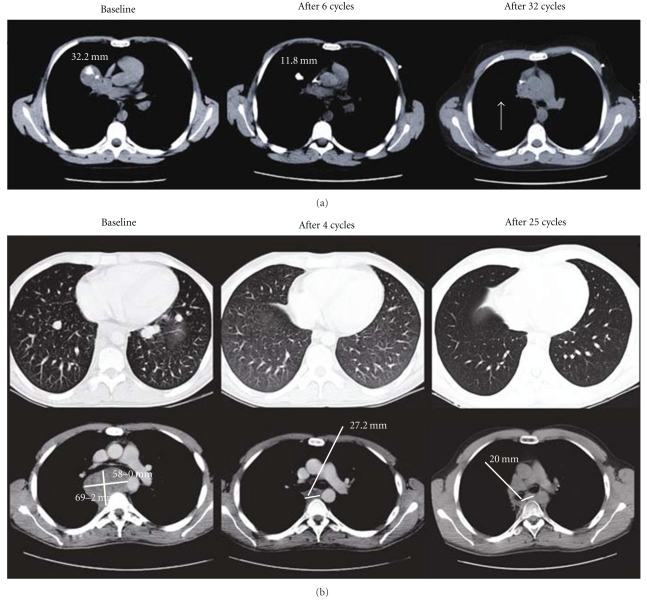
Confirmed responses to figitumumab in Ewing's sarcoma. (a) This figure illustrates a response in a 12-year-old male patient with metastatic Ewing's sarcoma treated with figitumumab 20 mg/kg every 4 weeks. The baseline, 6 and 32 cycle CT scans show a complete response (confirmed pathologically) in the target hilar mass and other subcentimeter lung nodules. (b) This figure illustrates a response in a 24-year-old male patient with metastatic extraskeletal Ewing's sarcoma treated with figitumumab 20 mg/kg every 4 weeks. The baseline and cycle 4 CT scan demonstrate complete eradication of several <2 cm lung metastases and a significant reduction in the mediastinal mass. The response to figitumumab was consolidated with 45 Gy in 15 fractions. The patient has an ongoing partial response after 25 cycles. (Adapted from [67]).

To date, three early studies involving the evaluation of IGF-1R mAbs in Ewing's sarcoma have been published. The larger study, by Olmos et al. [67], enrolled 29 patients with sarcoma, of which 15 had refractory Ewing's sarcoma. These patients were treated with figitumumab at the recommended dose of 20 mg/kg every four weeks. These patients were heavily pretreated (median of 3 lines), and notably 6 adolescent/paediatric patients (over 12 years of age) were included in Ewing's sarcoma expansion cohort. Fourteen Ewing's sarcoma patients were evaluable for radiological response, and 2 durable and ongoing radiological objective responses were observed, which included a pathological complete response (CR) (currently, 36+ months) in a 12 year old male, and a partial response (PR) (currently, 23+ months) in a young adult male (both responses are illustrated in Figure 2). In addition, 6 and 4 Ewing's sarcoma patients were free of disease progression at 3 and 6 months, respectively. Furthermore, five of these Ewing's sarcoma patients with prolonged stable disease (SD) had shrinkage of the target tumour lesions. Overall, the nonprogression rate at 3 months was 53% (CI-95% 28–78) and at 6 months was 40% (CI-95% 15–65) for all Ewing's sarcoma patients included in the study. However, as this was a Phase I expansion cohort, it was not powered to formally detect antitumour activity as a primary endpoint [67].

The second Phase I study, reported by Tolcher et al. [75], studied the mAb ganitumab. This study included 12 adult Ewing's sarcoma patients who were treated with doses of 12 and 20 mg/kg every 2 weeks. Ewing's sarcoma patients received ganitumab on days 1, 15, and 29; and this was followed by a 28-day treatment-free period before resuming the drug if tumour response was observed. One patient with Ewing's sarcoma attained a radiological CR which was maintained for 30 months. An additional Ewing's sarcoma patient achieved an unconfirmed PR but was withdrawn from the study due to a myelodysplastic syndrome (non ganitumab related). No other objectives responses or prolonged disease stabilisation were reported [75]. 

A third mAb, R1507, has shown promising preliminary activity in Ewing's sarcoma. The Phase I study of a weekly schedule of R1507 enrolled 9 Ewing's sarcoma patients [73]. These patients were treated with doses ranging from 1 mg/kg to 9 mg/kg weekly. Two Ewing's sarcoma patients had durable PRs (lasting 11 and 26+ months), and a further 2 had SD lasting for 4.3 and 6 months respectively. 

Finally, a preliminary report of SCH-717454 was presented by Anderson et al. in the 2008 Annual Connective Tissue Oncology Society (CTOS) Meeting [66]. This study demonstrated radiological responses in patients with Ewing's sarcoma [66]. This ongoing study included patients with refractory/resistant Ewing's sarcoma, as well as patients with other sarcoma subtypes who were treated at a dose of 9 mg/kg every week.

### 5.2. Phase II Studies with Anti-IGF-1R mAbs Involving Ewing Sarcoma Patients

The exciting preliminary results with anti-IGF-1R mAbs led to the development of a Phase II study in a variety of sarcoma subtypes, including Ewing's sarcoma, conducted by the Sarcoma Alliance for Research through Collaboration (SARC) study group. This ambitious study had 5 arms for specific sarcoma subtypes and had a planned recruitment of approximately 300 patients. The results of this study were reported during the 2010 American Society of Clinical Oncology (ASCO) annual meeting [74]. A Green and Dahlberg two-stage design was employed and the study included 111 Ewing's sarcoma patients from 30 centres across North America and Europe. Patients were treated with 9 mg/kg weekly of R1507 and stratified in two different cohorts at study entry: poor prognosis cohort (relapse/refractory disease <24 months and/or ≥2 chemotherapy regimens) which included 67 patients and a good prognosis cohort (relapse ≥24 months and <2 prior chemotherapy regimens) which included 44 patients. A total of 10 confirmed objective responses were observed using WHO criteria [90], 1 CR, and 9 PRs. A further 7 patients achieved unconfirmed partial responses but progressed rapidly after the first radiological evaluation. Objective responses were equally distributed between the poor and good prognosis cohorts (approximately 9% in both). The median duration of response in these patients was 25 weeks (range 12–47). A further 17 patients had confirmed SD as the best response, 3 of these would have been defined as PRs if RECIST rather than WHO criteria had been employed [91]. The median overall survival (OS) for patients treated in this study was 6.9 months.

A Phase II study of the IGF-1R mAb, ganitumab (AMG479), in Ewing's sarcoma and desmoplastic small round cell tumour (DSRCT) patients was also presented at the 2010 ASCO annual meeting [76]. The principal objective of this study was to determine the objective response rate (ORR) in patients who had not received prior therapy with an IGF-1R inhibitor; however, there was an exploratory cohort evaluating patients who had previously received another anti-IGF-1R targeted therapy. All patients received ganitumab at 12 mg/kg every 2 weeks. A total of 19 Ewing's sarcoma patients entered the primary cohort, and 3 were recruited to the exploratory cohort (no further data are currently available). One Ewing's sarcoma patient attained a PR, and a further 7 Ewing's patients achieved SD as best response; however, only one of these remained progression free beyond 24 weeks. The median progression-free survival (PFS) for Ewing's sarcoma patients included in this trial was 7.9 weeks.

More recently, Juergens et al. [68] presented the preliminary results of a Phase 2 study of figitumumab in paediatric (10 years or older) and adult patients with refractory Ewing's sarcomas. In this study, 106 patients were evaluable for objective response (RECIST), 15 patients had PRs, and 25 had stable disease. The median PFS for the overall population was poor 1.9 months (CI-95% 1.8–2.1), and the median overall survival was 8.9 months (CI-95% 7.2–10.8). However, in those patients with elevated blood IGF-1 levels (>110 ng/mL) at baseline, there was a significant advantage (*P* < .001) in OS compared with those with low IGF-1 (<110 ng/mL), that is, 10.5 months and 4.5 months, respectively. 

To our knowledge, there are two further Phase II studies of IGF1-R inhibition in Ewing's: (1) a study of SCH717454 in Ewing's sarcoma and osteosarcoma patients ≥4 years of age (http://www.clinicaltrials.gov/, NCT00617890) has a planned recruitment of 190 patients and (2) a study of cixutumumab (http://www.clinicaltrials.gov/, NCT00668148) in 185 patients (≥12 years) and fivearms: Ewing's sarcoma, rhabdomyosarcoma, leiomyosarcoma, adipocytic sarcomas and synovial sarcoma. 

Despite the preclinical data and promising early clinical results in Ewing's sarcoma, the recent Phase II results with anti-IGF-1R mAb as monotherapy have been less impressive than initially hoped (Table 2). Preliminary data for the mTOR inhibitor, radiforolimus (previously known as deferolimus), have shown a nonprogression rate of 30% at 16 weeks in bone sarcomas [21]. The mTOR inhibitor was deemed active and a Phase III trial comparing radiforolimus with placebo, as maintenance therapy, has recently completed recruitment. Other targeted agents have also been explored, and in a recent trial of imatinib in various sarcoma subtypes, no clinical activity was seen in patients with Ewing's sarcoma [92].

The results of Phase II studies published to date have been disappointing, and the clinical development pathway for this class of agents is currently very uncertain. Furthermore, the poor results observed with these agents in lung cancer have led to Roche halting further development of R1507 in all tumours [93, 94]. However, there is still the promise that these agents may have a role in the management of Ewing's sarcoma, either as monotherapy in selected patients or in combination regimens.

### 5.3. Toxicity with IGF-1R Monoclonal Antibodies

In general, IGF-1R mAbs are well tolerated, with the most common toxicities being mild and occasionally moderate. Severe (grade 3) or life-threatening (grade 4) adverse events are rare. Potential grade 3 and 4 hematologic adverse events reported in the Phase II trial with ganitumumab and R1507 included thrombocytopenia [74, 76], anemia [74, 76], neutropenia [76], pain at the time of administration [74], hyponatremia [74], and hyperglycemia [74, 76]. Thrombocytopenia was also reported in Phase I studies [69, 73, 75]. Grade 3 and 4 nonhematologic adverse events with figitumumab in sarcoma patients included deep venous thrombosis (*n* = 1), vomiting (*n* = 1), and back pain (*n* = 1). Grade 3 fatigue was also reported with figitumumab in nonsarcoma patients [95, 96]. Other relevant grade 3-4 nonhematological adverse events described with other IGF-1R mAbs include fatigue [72, 75, 97], arthralgia [75], chills [72], pneumonitis [69], nausea or vomiting [72], rash and/or pruritus [72], pain [66, 72], and gastrointestinal bleeding [69]. 

Hyperglycaemia is a common toxicity of all the mAbs, with grade 3 hyperglycaemia seen in several studies [66, 72, 97]. The mechanism for hyperglycaemia is unclear although IGF-1R may be involved in glucose metabolism via crosstalk and heterodimerisation with the insulin receptor [98–101]. This observation, and the increased plasma insulin levels reported after treatment with IGF-1R mAbs [96, 102], suggests compensatory insulin secretion and associated insulin resistance, the latter possibly secondary to increased IGF-1 and growth-hormone levels [82, 103]. Other severe laboratory abnormalities observed in sarcoma patients include uric acid elevation and transaminitis [67].

Interestingly, despite the expression of IGF-1R in vascular smooth muscle and endothelial cells [104] and the potential cardiotoxicity associated with mAbs, no cardiac toxicity has been reported to date. In the case of sarcoma patients treated with figitumumab, it is noteworthy that three-quarters of the patients were pretreated with anthracyclines and none developed cardiotoxicity [67].

Theoretically, IGF-1R mAbs would be expected to have an inhibitory effect on IGF and growth hormone-mediated growth. Thus, IGF-1R blockade could cause linear and somatic growth delay in a childhood and teenage population, as supported by the identification of patients with genetic defects in the IGF-1 axis such as IGF-1 deficiency [105]. This potential long-term adverse event is extremely important in the management of young sarcoma patients [1]. The current clinical experience is too limited to definitively address this question [67]. Detailed assessments of growth and hormone levels have been included in ongoing Phase II trials recruiting paediatric and prepubertal teenage patients, and it is hoped that these studies will provide insights to the effect of IGF-1R targeted therapy on growth during childhood and puberty.

### 5.4. Early Experience with Tyrosine Kinase Inhibitors of IGF1-R

There are a number of small molecule tyrosine kinase inhibitors (TKIs) of IGF1-R that are currently being, or have been, evaluated (Table 1). Some of these small molecules also inhibit IR-A, a component of IGF-R hybrid receptors [83]. Although this can potentially result in greater antitumour activity, it may also be associated with a higher incidence of metabolic toxicity. From the results of clinical trials of monoclonal antibodies and tyrosine kinase inhibitors in other tumour types, it is apparent that predicting differences in efficacy between these two classes can be difficult [106]. Notably, small molecule tyrosine kinase inhibitors do not directly activate the immune response against tumour cells, but they may be more effective when activated receptors are localised in cytoplasmic caveosomes and/or endosomes.

Some of these novel IGF-1R TKIs (i.e., picropodophylin (PPP), GSK183870A, GSK1904529A, BMS-536924, NVP-AEW541) have already shown promising preclinical activity as single agents or in combination in different sarcoma models [50, 51, 53, 79, 81, 107–109]. At the present time, only OSI-906 has been tested in Ewing's sarcoma patients (*n* = 2) although no antitumor activity was seen in these two cases [77]. However, currently there are insufficient data to define any difference in clinical benefit in patients treated with these two classes of IGF1-R inhibitors.

### 5.5. Combination Therapy with IGF-1R mAbs

IGF-1R activation has been associated with chemoresistance in multiple cancers [110], including some sarcomas such as Ewing's sarcoma [39]. Indeed, modulation of IGF signalling has been shown to enhance the antitumor activity of cytotoxic drugs in laboratory sarcoma models [58]. Thus, a strategy based on the combination of first- or second-line sarcoma chemotherapy with IGF-1R mAbs seems a rational approach in the utilisation of these agents. Currently, there are a number of ongoing or planned studies evaluating such combinations, including a Phase I/II trial of cixutumumab in combination with doxorubicin for advanced and unresectable soft-tissue sarcomas (http://www.clinicaltrials.gov/, NCT00720174), sponsored by the National Cancer Institute and a Phase I of SCH-717454 in combination with different commonly used chemotherapies in sarcoma such as vincristine, doxorubicin, and cyclophosphamide (CAV) or ifosfamide and etoposide (http://www.clinicaltrials.gov/, NCT00960063). 

Furthermore, clinical studies of rational combinations of IGF-1R mAbs with other targeted therapies are in progress. Examples of such regimens are the use of mTOR inhibitors in combination with IGF-1R antibodies [49, 111]. Studies evaluating this approach include a trial of RAD001 (everolimus) in combination with figitumumab sponsored by the Dana-Faber Cancer Institute [112]. This study enrolled a total of 21 sarcoma patients one of whom had Ewing's sarcoma. The reported toxicity profile for this combination was not significantly different from that of single agent everolimus. Grade 3 toxicity occurred in ≤10% of patients, and included mucositis, nausea, vomiting, and diarrhoea. One patient with Ewing's sarcoma maintained stable disease for six months. In addition, a trial of temsirolimus with cixutumumab (http://www.clinicaltrials.gov/, NCT01016015) is actively recruiting sarcoma patients. Other rational combinations could include regimens with heat shock protein 90 [113] or EGFR/HER2 inhibitors [107], as these have been implicated in potential mechanisms of resistance to IGF-1R inhibition in sarcoma cell lines.

### 5.6. Patient Selection

Despite robust preclinical evidence supporting the role of IGF1-R-targeted agents in Ewing's sarcoma, clinical results show that only a proportion of patients derive significant benefit, with many progressing early, even after an initial response. Although initial reports suggested an association between the *EWS/FLI-1* type 1 translocation and response in Ewing's sarcoma [75], the purported predictive value of translocation type has not been observed consistently [67, 74, 76]. Clinical data in nonsmall cell lung cancer patients have suggested that circulating free IGF-1 may identify patients who derive clinical benefit from figitumumab [114]. Similar data has also been reported in the Phase II trial of figitumumab in refractory Ewing's sarcoma, in which patients with elevated IGF-1 at baseline achieved longer OS [68]. However, it still remains unclear if an elevated IGF-1 level at baseline is a predictive factor for response to IGF-1R antibodies or simply a prognostic factor. Nonetheless, as IGF system and the activation of the IGF1-R are complex, response and resistance mechanisms are unlikely to be entirely dependent on or explained by circulating IGF-1 [115, 116].

## 6. Conclusions

During the last two decades, large amounts of preclinical data have been accumulated supporting the use of agents targeting IGF-1R in Ewing's sarcoma. This rationale has been reinforced by the early reports of clinical activity with several IGF-1R antibodies in this disease. However, the benefit of this therapeutic approach clearly does not extend to all patients, with Phase II studies demonstrating less promising responses than initially anticipated. In addition to the exploration of IGF-1R in combination with chemotherapy and other targeted agents, there is an urgent need to identify predictive biomarkers to improve patient selection, as well as to elucidate the mechanisms of resistance to these drugs, thereby facilitating the development of rational combination regimens. Despite the disappointing Phase II data, this novel group of drugs does constitute an active treatment in a proportion of Ewing's sarcoma patients.

## Figures and Tables

**Table 1:** IGF pathway targeting strategies in development.

Name	Class	Route	Company	Phase	Remarks in Ewing sarcomas	References
*Anti-IGF 1-receptor monoclonal antibodies*						

AVE1642	Humanized IgG1	IV	Sanofi-Aventis	I-II	(i) Nil.	[65]
SCH-717454	Fully human IgG1	IV	Schering Plough	II	(i) PRs seen in EWS patients. (ii) Phase II in relapsed EWS or osteosarcoma.	[66]
CP-751,871 figitumumab	Fully human IgG2	IV	Pfizer	II-III	(i) CR and PR in 2 pts with EWS. Prolonged SD in patients with EWS, synovial sarcoma and fibrosarcoma. (ii) Phase II trial in refractory paediatric and adult EWS: 15 PRs and 21 SD by RECIST.	[67, 68]
IMC-A12 cixutumumab	Fully human IgG1	IV	ImClone systems	I-II	(i) Preclinical activity in EWS models. (ii) Phase II with 5 tiers: one for EWS.	[69, 70]
BIIB022	Fully human IgG4	IV	Biogen Idec	I	(i) Phase I dose-escalation ongoing in all solid tumors, no EWS enrolled yet.	[71]
MK-0646	Humanized IgG1	IV	Merck	II	(i) Phase I dose-escalation studies completed. (ii) SD in 2 patients: EWS and DSRCT respectively.	[69, 72]
R1507 Robatumumab	Fully human IgG1	IV	Hoffman-La Roche	II	(i) Several PRs and prolonged SD in pts with EWS. (ii) Phase II in multiple sarcoma subtypes halted since 12/22/2009.	[73, 74]
AMG-479	Fully human IgG1	IV	Amgen	II	(i) CR and PR in 2 EWS patients. (ii) Phase II in relapsed Ewing and DSRCT. In EWS 1 PR and 1 SD >6 months.	[75, 76]

*Anti-IGF-1-receptor small molecule/tyrosine kinase Inhibitors*						

INSM-18	Small molecule TKI	PO	Insmed	I-II	(i) An ATP competitive and reversible TKI, which also inhibits HER2.	
BMS-754807	Small molecule TKI	PO	Bristol Myers Squibb	I	(i) An ATP competitive and reversible TKI. (ii) In vitro activity against in EWS, RMS, liposarcoma cell lines.	[52]
OSI-906	Small molecule TKI	PO	OSI pharmaceuticals	I	(i) An ATP competitive and reversible TKI. (ii) Phase I enrolled 2 Ewing sarcoma patients without benefit.	[77]
XL-228	Small molecule TKI	IV	Exelixis	I	(i) An ATP competitive TKI of IGF-1R, Aurora, FGFR and Src. (ii) Prolonged SD in 2 pts with leiomyosarcoma (≥7 m) and liposarcoma (≥5 m) respectively.	[78]
BVP-51004 cyclolignan, PPP	Small molecule TKI	PO	Biovitrum	I	(i) A non-ATP competitive TKI. (ii) In vitro activity in multiple sarcoma resistant cell lines, including osteosarcoma.	[79]
A-947864	Small molecule TKI	PO	Abbott	Preclinical	(i) An ATP competitive TKI.	
BMS-554417	Small molecule TKI	PO	Bristol Myers Squibb	Preclinical	(i) An ATP-competitive and reversible TKI. (ii) In vitro activity in EWS and osteosarcoma cell lines.	[80]
NVP-AEW541 NVP-ADW742	Small molecule TKIs	PO	Novartis	Preclinical	(i) ATP competitive and reversible TKIs. (ii) In vitro and in vivo activity in EWS.	[51, 53]
GSK1904529A GSK1838705A	Small molecule TKIs	PO	GlaxoSmithKline	Preclinical	(i) ATP-competitive, reversible, TKIs of IGF-1R and IR. (ii) In vitro activity in EWS cell lines.	[50, 81]
AG1024 (Tyrphostin)	Small molecule TKI	N.A.	Merck	Preclinical	(i) Used mainly in preclinical drug testing, Non-ATP competitive.	

Ref: reference; IgG: immunoglobulin G; SD: stable disease; PR: partial response; CR: complete response; pt: patient; EWS: Ewing sarcoma; ESFTs: Ewing sarcoma family of tumors; RMS: rhabdomyosarcoma; DSRCT: desmoplastic small round cell tumor; TKI: tyrosine kinase inhibitor; m: months.

**Table 2:** Responses in clinical trials.

Drug	*N*	CR	PR	SD
*Figitumumab*				

Phase I [67]	15	1 (7%)	1 (7%)	6 (40%)
Phase II [68]	106	0	15 (14%)	25 (24%)

*R1507 Phase I*				

Phase I [73]	9	0	2 (22%)	2 (22%)
Phase II [74]	111	1 (1%)	7 (6%)	17 (15%)

*Ganitumumab*				

Phase I [75]	12	1 (8%)	1 (8%)	NA/NR
Phase II [76]	19	0	1 (5%)	7 (37%)

*N* = number of Ewing's patients; = confirmed complete response; PR: confirmed partial responses; SD: stable disease (best response); NA/NR: nonavailable/nonreported.

## References

[1] Horner MJ, Krapcho M, Neyman N (2009). *SEER Cancer Statistics Review, 1975–2006*.

[2] Miller RW, Young JL, Novakovic B (1995). Childhood cancer. *Cancer*.

[3] Fletcher CDM, Mertens F (2002). *World Health Organisation Classification of Tumours: Pathology and Genetics of Tumours of Soft Tissue and Bone*.

[4] Patel SJ, Lynch JW, Johnson T (2002). Dose-intense ifosfamide/doxorubicin/cisplatin based chemotherapy for osteosarcoma in adults. *American Journal of Clinical Oncology*.

[5] Hensley ML, Maki R, Venkatraman E (2002). Gemcitabine and docetaxel in patients with unresectable leiomyosarcoma: results of a phase II trial. *Journal of Clinical Oncology*.

[6] Grosso F, Jones RL, Demetri GD (2007). Efficacy of trabectedin (ecteinascidin-743) in advanced pretreated myxoid liposarcomas: a retrospective study. *Lancet Oncology*.

[7] Bernstein M, Kovar H, Paulussen M (2006). Ewing’s sarcoma family of tumors: current management. *Oncologist*.

[8] Parkin DM, Stiller CA, Nectoux J (1993). International variations in the incidence of childhood bone tumours. *International Journal of Cancer*.

[9] Jawad MU, Cheung MC, Min ES, Schneiderbauer MM, Koniaris LG, Scully SP (2009). Ewing sarcoma demonstrates racial disparities in incidence-related and sex-related differences in outcome: an analysis of 1631 cases from the SEER database, 1973–2005. *Cancer*.

[10] Balamuth NJ, Womer RB (2010). Ewing’s sarcoma. *The Lancet Oncology*.

[11] Nesbit ME, Gehan EA, Burgert EO (1990). Multimodal therapy for the management of primary, nonmetastatic Ewing’s Sarcoma of bone: a long-term follow-up of the first intergroup study. *Journal of Clinical Oncology*.

[12] Barker LM, Pendergrass TW, Sanders JE, Hawkins DS (2005). Survival after recurrence of Ewing’s sarcoma family of tumors. *Journal of Clinical Oncology*.

[13] Leavey PJ, Mascarenhas L, Marina N (2008). Prognostic factors for patients with Ewing sarcoma (EWS) at first recurrence following multi-modality therapy: a report from the children’s oncology group. *Pediatric Blood and Cancer*.

[14] Jurgens H, Ranft A, Dirksen U (2007). Risks of recurrence and survival after relapse in patients with Ewing tumor. *ASCO Meeting Abstracts 10012*.

[15] Delattre O, Zucman J, Plougastel B (1992). Gene fusion with an ETS DNA-binding domain caused by chromosome translocation in human tumours. *Nature*.

[16] Scotlandi K, Benini S, Sarti M (1996). Insulin-like growth factor I receptor-mediated circuit in Ewing’s sarcoma/peripheral neuroectodermal tumor: a possible therapeutic target. *Cancer Research*.

[17] Prieur A, Tirode F, Cohen P, Delattre O (2004). EWS/FLI-1 silencing and gene profiling of Ewing cells reveal downstream oncogenic pathways and a crucial role for repression of insulin-like growth factor binding protein 3. *Molecular and Cellular Biology*.

[18] Pollak MN, Chapman JW, Pritchard KI (2008). NCIC-CTG MA14 Trial: tamoxifen (tam) vs. tam + octreotide (oct) for adjuvant treatment of stage I or II postmenopausal breast cancer. *Journal of Clinical Oncology*.

[19] Schreiber I, Buchfelder M, Droste M (2007). Treatment of acromegaly with the GH receptor antagonist pegvisomant in clinical practice: safety and efficacy evaluation from the German Pegvisomant Observational Study. *European Journal of Endocrinology*.

[20] Goya M, Miyamoto S, Nagai K (2004). Growth inhibition of human prostate cancer cells in human adult bone implanted into nonobese diabetic/severe combined immunodeficient mice by a ligand-specific antibody to human insulin-like growth factors. *Cancer Research*.

[21] Chawla SP, Tolcher AW, Staddon AP (2007). Survival results with AP23573, a novel mTOR inhibitor, in patients (pts) with advanced soft tissue or bone sarcomas: update of phase II trial. *Journal of Clinical Oncology*.

[22] Olmos D, Tan DSW, Jones RL, Judson IR (2010). Biological rationale and current clinical experience with anti-insulin-like growth factor 1 receptor monoclonal antibodies in treating sarcoma: twenty years from the bench to the bedside. *Cancer Journal*.

[23] Jones JI, Clemmons DR (1995). Insulin-like growth factors and their binding proteins: biological actions. *Endocrine Reviews*.

[24] Nakae J, Kido Y, Accili D (2001). Distinct and overlapping functions of insulin and IGF-I receptors. *Endocrine Reviews*.

[25] De Meyts P, Whittaker J (2002). Structural biology of insulin and IGF1 receptors: implications for drug design. *Nature Reviews Drug Discovery*.

[26] Brogiolo W, Stocker H, Ikeya T, Rintelen F, Fernandez R, Hafen E (2001). An evolutionarily conserved function of the drosophila insulin receptor and insulin-like peptides in growth control. *Current Biology*.

[27] Dong MQ, Venable JD, Au N (2007). Quantitative mass spectrometry identifies insulin signaling targets in C. elegans. *Science*.

[28] Ryan PD, Goss PE (2008). The emerging role of the insulin-like growth factor pathway as a therapeutic target in cancer. *Oncologist*.

[29] Van Der Geer P, Hunter T, Lindberg RA (1994). Receptor protein-tyrosine kinases and their signal transduction pathways. *Annual Review of Cell Biology*.

[30] Ullrich A, Schlessinger J (1990). Signal transduction by receptors with tyrosine kinase activity. *Cell*.

[31] Chitnis MM, Yuen JSP, Protheroe AS, Pollak M, Macaulay VM (2008). The type 1 insulin-like growth factor receptor pathway. *Clinical Cancer Research*.

[32] Kuemmerle JF (2003). IGF-I elicits growth of human intestinal smooth muscle cells by activation of PI3K, PDK-1, and p70S6 kinase. *American Journal of Physiology*.

[33] Shelton JG, Steelman LS, White ER, McCubrey JA (2004). Synergy between PI3K/Akt and Raf/MEK/ERK pathways in IGF-1R mediated cell cycle progression and prevention of apoptosis in hematopoietic cells. *Cell Cycle*.

[34] Aleman IT (2005). Role of insulin-like growth factors in neuronal plasticity and neuroprotection. *Advances in Experimental Medicine and Biology*.

[35] Scotlandi K, Picci P (2008). Targeting insulin-like growth factor 1 receptor in sarcomas. *Current Opinion in Oncology*.

[36] Gorlick R, Anderson P, Andrulis I (2003). Biology of childhood osteogenic sarcoma and potential targets for therapeutic development: meeting summary. *Clinical Cancer Research*.

[37] Giustina A, Mazziotti G, Canalis E (2008). Growth hormone, insulin-like growth factors, and the skeleton. *Endocrine Reviews*.

[38] Schouten-van Meeteren AYN, Van Valk PD, Van Der Linden HC (2001). Insulin-like growth factor type 1 (IGF-1) and igf binding protein-3 in patients with ewing sarcoma family of tumors. *Cancer*.

[39] Yee D, Favoni RE, Lebovic GS (1990). Insulin-like growth factor I expression by tumors of neuroectodermal origin with the t(11;22) chromosomal translocation. A potential autocrine growth factor. *Journal of Clinical Investigation*.

[40] Strammiello R, Benini S, Manara MC (2003). Impact of IGF-I/IGF-IR circuit on the angiogenetic properties of Ewing’s sarcoma cells. *Hormone and Metabolic Research*.

[41] Mitsiades CS, Mitsiades NS, McMullan CJ (2004). Inhibition of the insulin-like growth factor receptor-1 tyrosine kinase activity as a therapeutic strategy for multiple myeloma, other hematologic malignancies, and solid tumors. *Cancer Cell*.

[42] Mateo-Lozano S, Tirado OM, Notario V (2003). Rapamycin induces the fusion-type independent downregulation of the EWS/FLI-1 proteins and inhibits Ewing’s sarcoma cell proliferation. *Oncogene*.

[43] Toretsky JA, Kalebic T, Blakesley V, LeRoith D, Helman LJ (1997). The insulin-like growth factor-I receptor is required for EWS/FLI-1 transformation of fibroblasts. *Journal of Biological Chemistry*.

[44] Riggi N, Stamenkovic I (2007). The Biology of Ewing sarcoma. *Cancer Letters*.

[45] Torchia EC, Jaishankar S, Baker SJ (2003). Ewing tumor fusion proteins block the differentiation of pluripotent marrow stromal cells. *Cancer Research*.

[46] De Alava E, Panizo A, Antonescu CR (2000). Association of EWS-FLI1 type 1 fusion with lower proliferative rate in Ewing’s sarcoma. *American Journal of Pathology*.

[47] Iwasa T, Okamoto I, Suzuki M (2009). Inhibition of insulin-like growth factor 1 receptor by CP-751,871 radiosensitizes non-small cell lung cancer cells. *Clinical Cancer Research*.

[48] Scotlandi K, Benini S, Nanni P (1998). Blockage of insulin-like growth factor-I receptor inhibits the growth of Ewing’s sarcoma in athymic mice. *Cancer Research*.

[49] Kurmasheva RT, Dudkin L, Billups C, Debelenko LV, Morton CL, Houghton PJ (2009). The insulin-like growth factor-1 receptor-targeting antibody, CP-751,871, suppresses tumor-derived VEGF and synergizes with rapamycin in models of childhood sarcoma. *Cancer Research*.

[50] Sabbatini P, Korenchuk S, Rowand JL (2009). GSK1838705A inhibits the insulin-like growth factor-1 receptor and anaplastic lymphoma kinase and shows antitumor activity in experimental models of human cancers. *Molecular Cancer Therapeutics*.

[51] Martins AS, Mackintosh C, Herrero Martín D (2006). Insulin-like growth factor I receptor pathway inhibition by ADW742, alone or in combination with imatinib, doxorubicin, or vincristine, is a novel therapeutic approach in Ewing tumor. *Clinical Cancer Research*.

[52] Carboni JM, Wittman M, Yang Z (2009). BMS-754807, a small molecule inhibitor of insulin-like growth factor-1R/IR. *Molecular Cancer Therapeutics*.

[53] Manara MC, Landuzzi L, Nanni P (2007). Preclinical in vivo study of new insulin-like growth factor-I receptor-specific inhibitor in Ewing’s sarcoma. *Clinical Cancer Research*.

[54] Beech DJ, Perer E, Helms J, Gratzer A, Deng N (2003). Insulin-like growth factor-I receptor activation blocks doxorubicin cytotoxicity in sarcoma cells. *Oncology Reports*.

[55] Sell C, Baserga R, Rubin R (1995). Insulin-like growth factor I (IGF-I) and the IGF-I receptor prevent etoposide-induced apoptosis. *Cancer Research*.

[56] Turner BC, Haffty BG, Narayanan L (1997). Insulin-like growth factor-I receptor overexpression mediates cellular radioresistance and local breast cancer recurrence after lumpectomy and radiation. *Cancer Research*.

[57] Manara MC, Perdichizzi S, Serra M (2005). The molecular mechanisms responsible for resistance to ET-743 (Trabectidin; Yondelis) in the Ewing’s sarcoma cell line, TC-71. *International Journal of Oncology*.

[58] Benini S, Manara MC, Baldini N (2001). Inhibition of insulin-like growth factor I receptor increases the antitumor activity of doxorubicin and vincristine against Ewing’s sarcoma cells. *Clinical Cancer Research*.

[59] Lu Y, Zi X, Zhao Y, Mascarenhas D, Pollak M (2001). Insulin-like growth factor-I receptor signaling and resistance to transtuzumab (Herceptin). *Journal of the National Cancer Institute*.

[60] Nahta R, Yuan LXH, Zhang B, Kobayashi R, Esteva FJ (2005). Insulin-like growth factor-I receptor/human epidermal growth factor receptor 2 heterodimerization contributes to trastuzumab resistance of breast cancer cells. *Cancer Research*.

[61] Thimmaiah KN, Easton J, Huang S (2003). Insulin-like growth factor i-mediated protection from rapamycin-induced apoptosis is independent of Ras-Erk1-Erk2 and phosphatidylinositol 3′-kinase-Akt signaling pathways. *Cancer Research*.

[62] Ullrich A, Gray A, Tam AW (1986). Insulin-like growth factor I receptor primary structure: comparison with insulin receptor suggests structural determinants that define functional specificity. *EMBO Journal*.

[63] Laviola L, Perrini S, Cignarelli A, Giorgino F (2006). Insulin signalling in human adipose tissue. *Archives of Physiology and Biochemistry*.

[64] Laviola L, Natalicchio A, Giorgino F (2007). The IGF-I signaling pathway. *Current Pharmaceutical Design*.

[65] Tolcher AW, Patnaik A, Till E (2008). A phase I study of AVE1642, a humanized monoclonal antibody IGF-1R (insulin like growth factor1 receptor) antagonist, in patients(pts) with advanced solid tumor(ST). *Journal of Clinical Oncology*.

[66] Anderson P, Skubitz K, Miller R, Meyer W, Lu B Activity of SCH 717454 in subjects with relapsed osteosarcoma or Ewing’s sarcoma (study P04720).

[67] Olmos D, Postel-Vinay S, Molife LR (2010). Safety, pharmacokinetics, and preliminary activity of the anti-IGF-1R antibody figitumumab (CP-751,871) in patients with sarcoma and Ewing’s sarcoma: a phase 1 expansion cohort study. *The Lancet Oncology*.

[68] Juergens H, Daw NC, Oberlin O (2010). Safety and efficacy results from a phase 1/2 study of the anti-IGF-1R antibody figitumumab in patients with refractory Ewing’s and other sarcomas. *Annals of Oncology*.

[69] Hidalgo M, Tirado Gomez M, Lewis N (2008). A phase I study of MK-0646, a humanized monoclonal antibody against the insulin-like growth factor receptor type 1 (IGF1R) in advanced solid tumor patients in a q2 wk schedule. *Journal of Clinical Oncology*.

[70] Kolb EA, Morton C, Houghton PJ (2008). Pediatric Preclinical Testing Program (PPTP) evaluation of the fully human anti-IGF-1R antibody IMC-A12. *European Journal of Cancer*.

[71] von Mehren M, Britten C, Lear K (2010). Phase I, dose-escalation study of BIIB022 (anti-IGF-1R antibody) in advanced solid tumors. *ASCO Meeting Abstracts 2612*.

[72] Atzori F, Tabernero J, Cervantes A (2008). A phase I, pharmacokinetic (PK) and pharmacodynamic (PD) study of weekly (qW) MK-0646, an insulin-like growth factor-1 receptor (IGF1R) monoclonal antibody (MAb) in patients (pts) with advanced solid tumors. *Journal of Clinical Oncology*.

[73] Kurzrock R, Patnaik A, Aisner J (2010). A phase I study of weekly R1507, a human monoclonal antibody insulin-like growth factor-I receptor antagonist, in patients with advanced solid tumors. *Clinical Cancer Research*.

[74] Pappo AS, Patel S, Crowley J (2010). Activity of R1507, a monoclonal antibody to the insulin-like growth factor-1 receptor (IGF1R), in patients (pts) with recurrent or refractory Ewing's sarcoma family of tumors (ESFT): results of a phase II SARC study. *ASCO Meeting Abstracts 10000*.

[75] Tolcher AW, Sarantopoulos J, Patnaik A (2009). Phase I, pharmacokinetic, and pharmacodynamic study of AMG 479, a fully human monoclonal antibody to insulin-like growth factor receptor 1. *Journal of Clinical Oncology*.

[76] Tap WD, Demetri GD, Barnette P (2010). AMG 479 in relapsed or refractory Ewing's family tumors (EFT) or desmoplastic small round cell tumors (DSRCT): phase II results. *ASCO Meeting Abstracts 10001*.

[77] Carden CP, Kim ES, Jones RL (2010). Phase I study of intermittent dosing of OSI-906, a dual tyrosine kinase inhibitor of insulin-like growth factor-1 receptor (IGF- 1R) and insulin receptor (IR) in patients with advanced solid tumors. *ASCO Meeting Abstracts 2530*.

[78] Smith DC, Britten C, Clary DO, Nguyen LT, Woodard P, Hurwitz HI (2009). A phase I study of XL228, a potent IGF1R/AURORA/SRC inhibitor, in patients with solid tumors or hematologic malignancies. *Journal of Clinical Oncology*.

[79] Duan Z, Choy E, Harmon D (2009). Insulin-like growth factor-I receptor tyrosine kinase inhibitor cyclolignan picropodophyllin inhibits proliferation and induces apoptosis in multidrug resistant osteosarcoma cell lines. *Molecular Cancer Therapeutics*.

[80] Haluska P, Carboni JM, Loegering DA (2006). In vitro and in vivo antitumor effects of the dual insulin-like growth factor-I/insulin receptor inhibitor, BMS-554417. *Cancer Research*.

[81] Sabbatini P, Rowand JL, Groy A (2009). Antitumor activity of GSK1904529A, a small-molecule inhibitor of the insulin-like growth factor-I receptor tyrosine kinase. *Clinical Cancer Research*.

[82] Pollak M (2008). Insulin and insulin-like growth factor signalling in neoplasia. *Nature Reviews Cancer*.

[83] Gualberto A, Pollak M (2009). Emerging role of insulin-like growth factor receptor inhibitors in oncology: early clinical trial results and future directions. *Oncogene*.

[84] Goetsch L, Gonzalez A, Leger O (2005). A recombinant humanized anti-insulin-like growth factor-receptor type I antibody (h7C10) enhances the antitumor activity of vinorelbine and anti-epidermal growth factor receptor therapy against human cancer xenografts. *International Journal of Cancer*.

[85] Burtrum D, Zhu Z, Lu D (2003). A fully human monoclonal antibody to the insulin-like growth factor I receptor blocks ligand-dependent signaling and inhibits human tumor growth in vivo. *Cancer Research*.

[86] Beltran PJ, Mitchell P, Chung YA (2009). AMG 479, a fully human anti-insulin-like growth factor receptor type I monoclonal antibody, inhibits the growth and survival of pancreatic carcinoma cells. *Molecular Cancer Therapeutics*.

[87] Anders Kolb E, Gorlick R, Houghton PJ (2008). Initial testing (stage 1) of a monoclonal antibody (SCH 717454) against the IGF-1 receptor by the pediatric preclinical testing program. *Pediatric Blood and Cancer*.

[88] Cohen BD, Baker DA, Soderstrom C (2005). Combination therapy enhances the inhibition of tumor growth with the fully human anti-type 1 insulin-like growth factor receptor monoclonal antibody CP-751,871. *Clinical Cancer Research*.

[89] Presta LG (2008). Molecular engineering and design of therapeutic antibodies. *Current Opinion in Immunology*.

[90] Miller AB, Hoogstraten B, Staquet M, Winkler A (1981). Reporting results of cancer treatment. *Cancer*.

[91] Therasse P, Arbuck SG, Eisenhauer EA (2000). New guidelines to evaluate the response to treatment in solid tumors. *Journal of the National Cancer Institute*.

[92] Chugh R, Wathen JK, Maki RG (2009). Phase II multicenter trial of imatinib in 10 histologic subtypes of sarcoma using a bayesian hierarchical statistical model. *Journal of Clinical Oncology*.

[93] http://www.reuters.com/article/idUSTRE5BS3XY20091229.

[94] http://www.sarctrials.org/public/press91.aspx.

[95] Haluska P, Worden F, Olmos D (2010). Safety, tolerability, and pharmacokinetics of the anti-IGF-1R monoclonal antibody figitumumab in patients with refractory adrenocortical carcinoma. *Cancer Chemotherapy and Pharmacology*.

[96] Haluska P, Shaw HM, Batzel GN (2007). Phase I dose escalation study of the anti-insulin-like growth factor-I receptor monoclonal antibody CP-751,871in patients with refractory solid tumors. *Clinical Cancer Research*.

[97] Higano CS, Yu EY, Whiting SH (2007). A phase I, first in man study of weekly IMC-A12, a fully human insulin like growth factor-I receptor IgG1 monoclonal antibody, in patients with advanced solid tumors. *Journal of Clinical Oncology*.

[98] Guler HP, Zapf J, Froesch ER (1987). Short-term metabolic effects of recombinant human insulin-like growth factor I healthy adults. *New England Journal of Medicine*.

[99] Clemmons DR (2006). Involvement of insulin-like growth factor-I in the control of glucose homeostasis. *Current Opinion in Pharmacology*.

[100] Moses AC, Young SCJ, Morrow LA, O’Brien M, Clemmons DR (1996). Recombinant human insulin-like growth factor I increases insulin sensitivity and improves glycemic control in type II diabetes. *Diabetes*.

[101] Pennisi P, Gavrilova O, Setser-Portas J (2006). Recombinant human insulin-like growth factor-I treatment inhibits gluconeogenesis in a transgenic mouse model of type 2 diabetes mellitus. *Endocrinology*.

[102] Lacy MQ, Alsina M, Fonseca R (2008). Phase I, pharmacokinetic and pharmacodynamic study of the anti-insulinlike growth factor type 1 receptor monoclonal antibody CP-751,871 in patients with multiple myeloma. *Journal of Clinical Oncology*.

[103] Del Rincon JP, Iida K, Gaylinn BD (2007). Growth hormone regulation of p85*α* expression and phosphoinositide 3-kinase activity in adipose tissue: mechanism for growth hormone-mediated insulin resistance. *Diabetes*.

[104] Delafontaine P, Song YH, Li Y (2004). Expression, regulation, and function of IGF-1, IGF-1R, and IGF-1 binding proteins in blood vessels. *Arteriosclerosis, Thrombosis, and Vascular Biology*.

[105] Laron Z, Anin S, Klipper-Aurbach Y, Klinger B (1992). Effects of insulin-like growth factor on linear growth, head circumference, and body fat in patients with Laron-type dwarfism. *Lancet*.

[106] Imai K, Takaoka A (2006). Comparing antibody and small-molecule therapies for cancer. *Nature Reviews Cancer*.

[107] Huang F, Greer A, Hurlburt W (2009). The mechanisms of differential sensitivity to an insulin-like growth factor-1 receptor inhibitor (BMS-536924) and rationale for combining with EGFR/HER2 inhibitors. *Cancer Research*.

[108] Friedrichs N, Küchler J, Endl E (2008). Insulin-like growth factor-1 receptor acts as a growth regulator in synovial sarcoma. *Journal of Pathology*.

[109] Scotlandi K, Manara MC, Nicoletti G (2005). Antitumor activity of the insulin-like growth factor-I receptor kinase inhibitor NVP-AEW541 in musculoskeletal tumors. *Cancer Research*.

[110] Weroha SJ, Haluska P (2008). IGF-1 receptor inhibitors in clinical trials—early lessons. *Journal of Mammary Gland Biology and Neoplasia*.

[111] Wan X, Helman LJ (2007). The biology behind mTOR inhibition in sarcoma. *Oncologist*.

[112] Quek R, Wang Q, Morgan JA (2011). Combination mTOR and IGF-1R inhibition: phase I trial of everolimus and figitumumab in patients with advanced sarcomas and other solid tumors. *Clinical Cancer Research*.

[113] Martins AS, Ordoñez JL, García-Sánchez A (2008). A pivotal role for heat shock protein 90 in Ewing sarcoma resistance to anti-insulin-like growth factor 1 receptor treatment: in vitro and in vivo study. *Cancer Research*.

[114] Gualberto A, Hixon ML, Karp DD (2011). Pre-treatment levels of circulating free IGF-1 identify NSCLC patients who derive clinical benefit from figitumumab. *British Journal of Cancer*.

[115] Olmos D, Basu B, De Bono JS (2010). Targeting insulin-like growth factor signaling: rational combination strategies. *Molecular Cancer Therapeutics*.

[116] Basu B, Olmos D, De Bono JS (2011). Targeting IGF-1R: throwing out the baby with the bathwater. *British Journal of Cancer*.

